# Fetal Nicotine Exposure Increases Preference for Nicotine Odor in Early Postnatal and Adolescent, but Not Adult, Rats

**DOI:** 10.1371/journal.pone.0084989

**Published:** 2013-12-17

**Authors:** Nicole M. Mantella, Paul F. Kent, Steven L. Youngentob

**Affiliations:** 1 Department of Psychiatry and Behavioral Sciences, State University of New York Upstate Medical University, Syracuse, New York, United States of America; 2 Department of Neurology, State University of New York Upstate Medical University, Syracuse, New York, United States of America; 3 State University of New York Developmental Exposure Alcohol Research Center, Syracuse and Binghamton, New York, United States of America; Columbia University, United States of America

## Abstract

Human studies demonstrate a four-fold increased possibility of smoking in the children of mothers who smoked during pregnancy. Nicotine is the active addictive component in tobacco-related products, crossing the placenta and contaminating the amniotic fluid. It is known that chemosensory experience in the womb can influence postnatal odor-guided preference behaviors for an exposure stimulus. By means of behavioral and neurophysiologic approaches, we examined whether fetal nicotine exposure, using mini-osmotic pumps, altered the response to nicotine odor in early postnatal (P17), adolescent (P35) and adult (P90) progeny. Compared with controls, fetal exposed rats displayed an altered innate response to nicotine odor that was evident at P17, declined in magnitude by P35 and was absent at P90 - these effects were specific to nicotine odor. The behavioral effect in P17 rats occurred in conjunction with a tuned olfactory mucosal response to nicotine odor along with an untoward consequence on the epithelial response to other stimuli – these P17 neural effects were absent in P35 and P90 animals. The absence of an altered neural effect at P35 suggests that central mechanisms, such as nicotine-induced modifications of the olfactory bulb, bring about the altered behavioral response to nicotine odor. Together, these findings provide insights into how fetal nicotine exposure influences the behavioral preference and responsiveness to the drug later in life. Moreover, they add to a growing literature demonstrating chemosensory mechanisms by which patterns of maternal drug use can be conveyed to offspring, thereby enhancing postnatal vulnerability for subsequent use and abuse.

## Introduction

 Although there are over 4000 different chemicals in inhaled cigarette smoke, nicotine is the addictive component and the key constituent responsible for the maintenance of tobacco product use (e.g.,[1]). Nicotine is a neurotoxin that crosses the blood-brain barrier. As such, fetal nicotine exposure can yield profound and permanent sequelae for the developing fetus. Importantly, rodent models of fetal nicotine exposure both parallel and predict findings from maternal smoking studies in humans (for rev. see 2).

 Prenatal exposure via maternal smoking can cause a variety of behavioral changes. These include but are not limited to hyperactivity, impulsivity, and defects in learning, memory and attention [3-7]. These behavioral effects are in addition to retarded fetal growth, premature birth, stillbirths and increased mortality of newborn [8-11].

 An additional untoward consequence of maternal smoking is a four-fold increased probability of smoking among a mother’s children [6,11,12]. In addition, these children initiate smoking at an earlier age [12] and have an enhanced probability both of adolescent abuse and adult addiction [12-14]. A fundamental question emerging from these observations is how does fetal exposure increase the risk of cigarette smoking and long-term abuse. Indeed, understanding the factors contributing to the fetal induced behavioral phenotype is critical for tobacco prevention and cessation treatment.

 There are noteworthy similarities between the untoward consequences in neural development associated with fetal nicotine exposure and those stemming from prenatal ethanol: encompassing the range of neurobehavioral and developmental effects noted above (see rev [15].). Notably, human clinical and epidemiologic studies of fetal ethanol exposure (like those reported for nicotine, above) demonstrate that fetal ethanol exposure: (a) enhances the probability of adolescent abuse; and (b) is the best predictor of adolescent and long-term abuse [16-19]. These parallel findings have potentially important implications for studies of fetal nicotine exposure. There is a large scientific literature related to what the fetus can learn during development about chemosensory stimuli, such as ethanol, contaminating the amniotic milieu. Using a rat model of prenatal ethanol experience, studies have demonstrated that such exposures resulted in: (1) alterations in the attentiveness to the odor of ethanol and enhanced odor preference (e.g., 20); (2) enhanced avidity for ethanol (e.g., 21,22); and (3) the capacity of ethanol to act as an associative cue (e.g., 23,24). Human experiments also show that prenatal exposure to ethanol alters postnatal orienting responses to ethanol odor [25].

 In extension to the above, studies demonstrate that chemosensory function influences the responsiveness to ethanol. Fetal ethanol experience resulted in increased ethanol intake in addition to the behavioral response to the drug’s odor in early postnatal rats [26,27]. This latter consequence occurred by way of a maternal ethanol treatment effect on the responsiveness of the olfactory epithelium [28]. Interestingly, these equivalent consequences were absent in adults (e.g., 26,28). The enhanced behavioral odor-mediated response, however, persisted into the age-range of adolescence [29,30].

 More general to the above points, there is unambiguous evidence that as a general phenomenon olfactory plasticity serves to focus an animal’s awareness or responsiveness to odorants that are assumed important. Indeed, studies demonstrate that from the fetal period through adulthood olfactory experience influences olfactory sensory function (e.g., 31-35). Nicotine, like ethanol and other chemosensory stimuli, is a substance capable of infiltrating the amniotic fluid via maternal use (e.g., 11,36). As such, the foregoing data discussion suggests that stimulus-induced olfactory plasticity, resulting from fetal nicotine exposure, may be important to the enhancements in nicotine avidity described in the human epidemiology. In the present study, we used behavioral and neurophysiologic methods to test whether prenatal nicotine experience altered the odor-mediated behavioral responsiveness to nicotine and, if so, whether such changes were paralleled by neurophysiologic alterations in the response of the olfactory epithelium in the early postnatal, adolescent and adult animal. In this latter regard we were particularly interested in testing whether any observable prenatal effect persisted into adulthood.

## Materials and Methods

### Ethics Statement

 All experiments and methods were sanctioned by SUNY Upstate Medical University’s Committee for the Humane use of Animals (PHS Assurance: A3514-01). Appropriate procedures were in place to minimize suffering where applicable.

### General

 We examined both the neurophysiologic along with the odorant-induced behavioral responses (i.e., sniffing airflow patterns) of prenatal nicotine or control exposed P17, P35 or P90 rats, using optical recording procedures and whole-body plethysmography. P17 was chosen because this age falls within a range of early ages that show an ability to recall a fetal chemosensory exposure (e.g., 23,24) and the effects of fetal experience-induced olfactory plasticity (e.g., 28) for another drug of abuse, namely, ethanol. P35 was chosen because it represents a time point roughly midway within adolescence (P28-P42: e.g., [37]). Further, studies examining the consequences of fetal ethanol experience demonstrate that the effects persist into adolescence (e.g., 29,30,38). Finally, assessing P90 animals (i.e., adults) permitted fuller evaluation of the ontogeny of any fetal nicotine effect. Moreover, it allowed us to test the hypothesis that the consequences of fetal nicotine exposure on olfactory function would ameliorate by adulthood.

### Prenatal Exposure Paradigm and Treatment of Dams

 Prenatal nicotine delivery (nicotine tartrate salt: Sigma Aldrich, Allentown, PA) was accomplished via a mini-osmotic pump surgically implanted into pregnant rat dams (e.g., 39,40). This approach had several advantages for our purpose. First, compared to repeated daily nicotine injections that yield spikes in plasma blood nicotine levels, the pump delivers the drug at a controlled rate over weeks [41,42]. Second, the pump method also avoided the stress of repeated injections and eliminated spikes in blood nicotine levels obviating the risk of hypoxic periods that can influence fetal brain development (e.g., 42). The mini-osmotic pump approach also allowed us to generate a comparatively constant exposure dose during prenatal olfactory system development: especially when the sensory neurons of the epithelium first begin to respond to odor stimuli in the rat fetus (gestational [G] day 14) [43] and just before their axons have reached the olfactory bulb around this same time (G14-15) [44].

 Pregnant Long-Evans rats (Harlan Laboratory, Indianapolis, IN) were weighed on G5 and then placed into triads of weight-matched groups. Within a weight-matched triad of dams the animals were then randomly assigned to the NIC (nicotine treated), VC (vehicle control) or NP (no surgery control) maternal treatment groups. In this latter regard, VC animals are the historical control of choice for any potential effect of “surgery” in studies using mini-osmotic pumps (e.g., 45-47). In the present study, we also included a non-surgical control in order to directly examine whether and to what degree the procedure of pump implantation, itself, had an effect on our dependent variables of interest.

 For the NIC and VC groups, respectively, nicotine or vehicle only loaded Azlet 2ML2 mini-osmotic pumps (Azlet, Cupertino, CA) were surgically implanted using aseptic procedures. All surgeries took place between 9 AM and noon on G7. Pumps were inserted through a 2-mm incision site made on the back of a shaved and aseptic prepped anesthetized dam (Fluothane). A 6-mm pocket for the pump was made with sterile forceps. Sterile wound clips were used to close the incision sites. The entire procedure from beginning of anesthesia to wound closure took less than ten minutes. All dams were maintained on standard lab chow and water throughout gestation.

### Nicotine Dose

 In the present study, we chose a target blood nicotine level indicative of a “typical” smoker, namely, approximately 25 ng/ml (e.g., 48,49). In this respect, despite the known influence of nicotine on both pre- and post-natal development there is relatively little data on the dose kinetics of nicotine in pregnant rats. Importantly, when using mini-osmotic pumps it must also be highlighted that independent of the dose of nicotine delivered, a linear relationship between dose delivered and blood nicotine level does not exist throughout pregnancy. In other words, the loading dose of the osmotic pump is based on the dam’s initial weight at the time of pump implantation, which of course is changing throughout pregnancy. Therefore, prior to initiation of the primary study of interest we conducted two pilot experiments.

 In the first pilot experiment, we conducted a study of dose-response. Guided by the work of Murrin and colleagues [39] and Hussein [50], osmotic pumps were implanted in pregnant dams on G7, as described above, with one of four nicotine concentrations (0.625, 1.25, 2.5 or 5.0 mg/kg/day: N= 5 dams/concentration). Dams were sacrificed on G19 (two days prior to delivery) and trunk blood collected. Blood nicotine levels were determined by high performance liquid chromatography in conjunction with tandem mass spectrometry (NMS Labs, Willow Grove, PA). On the basis of this study, 5.0mg/kg/day proved to be the appropriate dose (see Results).

 In pilot experiment two, we determined the consistency of dose delivery across gestation. A total of eighteen pregnant dams were implanted on G7 and then randomly sacrificed on G10, G13, or G16 (N=5-6 per time point). Blood nicotine levels were determined, as above.

### Experimental Subjects

 Within 24 hours of the dams delivering, litters were reduced to 10 (with as close a male/female balance as possible) and fostered to dams specifically bred for this purpose. To prevent increasing the likelihood of a Type I error only one male and one female rat per litter was randomized to any testing age or study. As such, three rats of each gender from each litter of NIC, VC or NP dams were used. These animals were further randomized to the P17, P35 or P90 age of assessment. Ten blocks of three dams (NIC, VC, and NP) were used for experiments in which each animal was assessed for its behavioral and neurophysiologic responses. Ten additional blocks were used for a behavioral control experiment that specifically tested the response to ethylacetoacetate (EA) (that is, a non-fetal-exposure odorant).

### Behavioral Assessment

 Assessing odorant responsiveness by evaluating the innate odorant-induced sniffing response (i.e., changes in airflow) has become a model unbiased technique for evaluating the attentiveness/response to odorant stimuli in rodents (both mice and rats) as young as neonates and into adulthood. The basic theory and practical details of the approach have been described (e.g., 27,28,51). Briefly, using whole-body plethysmography, we monitored the respiratory airflow patterns (i.e. sniffing) in response to the delivery of a blank stimulus (i.e., air) or different concentrations of odorant into a testing chamber through which continuous airflow can be passed. A PC controlled both the parameters for behavioral testing and the production of odorant stimuli, as well as respiratory data acquisition.

 We recorded the innate stimulus-induced behavioral response to nicotine odor of male and female NIC, VC and NP rats of a particular age. Animal testing order was randomized. Following forty habituation trials consisting of only the presentation of air, air and nicotine odor were delivered randomly in 20 trial sets (10 air and 10 odorant). Using a five concentration ascending series (3.125x10^-3^, 6.25x10^-3^, 1.25x10^-2^, 2.5x10^-2^ and 5x10^-2^: fraction of vapor saturation at 20°C), each odorant concentration was separately presented for 1 set of 20 trials (e.g., 28). After testing, the rats were euthanized and the response to odorant stimulation of their septal olfactory epithelium assessed.

 In a separate set of rats, we behaviorally assessed the odorant EA (no neurophysiology was performed on these animals). This odorant was chosen based on prior studies of prenatal ethanol exposure [28]. Assessment of the odor-mediated behavioral response to EA tested for the specificity of the fetal nicotine exposure effect on the behavioral response to nicotine odor. The concentration series for EA was: 3.125x10^-3^, 6.25x10^-3^, 1.25x10^-2^, 2.5x10^-2^ and 5x10^-2^ (fraction of vapor saturation at 10°C). 

 The behavioral analysis proceeded according to our previously established approach (e.g., 27-30,52). The basis for our approach was based on several key prior findings. First, airflow patterns generated by sniffing behavior represent a response with a large number of interrelated variables that change both with different odorants and different concentrations of odorant [53]. Nonetheless, although these patterns can be broken down into a sizeable amount of descriptive quantitative attributes, information from any individual variable (e.g., number of inspiratory sniffs) is inadequate to evaluate the significance of an animal’s behavioral reaction to an odorant [51,53]. In contrast, sniffing behavior, as a totality, can be effectively evaluated by utilizing a number of deconstructed variables from the patterns along with their corresponding weightings (e.g., 28-30,51,53). These variables, in turn, can be used to generate an “Index” that quantifies the animals’ behavioral responses with a single measure that can be used to provide valid estimates of main effects (ibid). For example, using this approach, elevated postnatal ethanol intake (resulting from fetal ethanol experience) was shown to be causally linked to an enhanced response to the odor of ethanol [27].

 To create an “Index”, the sniffing pattern recorded for each stimulus presentation was first broken down by computer analysis into 14 respiratory response measures (in other words, behavioral response dimensions) (e.g., 28-30,52). The 14 response measures were: sniff frequency; the number of inspiratory and expiratory sniffs; the duration, volume, average flow rate, and peak flow rate of an inspiratory and expiratory sniff; the total inspiratory and expiratory volume; and the total apneic duration. Thus, each NIC, VC and NP animal at each of the P17, P35 and P90 ages contributed a set of 14 response variables at each of the five different odorant concentrations evaluated to the overall data matrix.

 Because the data set was multivariate, we performed a standard principle components analysis (PCA) on the entire data set in order to compress the deconstructed variables for each stimulus response of an animal into two uncorrelated values (i.e., factor 1 and 2 of the PCA) (*a priori* we focused our evaluations on those PCA factors with Eigen values above the Kaiser criterion of 1). In short, each animal’s 14 x 5 data matrix was reduced to a 2 x 5 matrix. In other words, the values of the two PCA factors for each odorant concentration defined an animal’s behavioral response to the stimulus (ibid).

 To generate a behavioral Index for each animal that integrated the rats’ responses across all stimulus concentrations separate multivariate linear regression analyses were performed on each PCA factor. For each analysis the 5 behavioral response values (one at each concentration) served as the dependent variables and maternal treatment as the independent variable. The results provided estimates of the coefficients for each concentration of odorant for the respective PCA factor. The final index value derived from each PCA factor for a rat was the arithmetic sum of the constant from the regression analysis, plus the respective PCA value at each odorant concentration multiplied by the estimated coefficient. This yielded x and y data pairs that located each NIC, VC, and NP animal in a behavioral response space.

 To formally test specific *a priori* hypotheses, univariate significance tests (two-tailed t: *P* < 0.05) were performed on the combined weighted city-block distance in terms of the effect sizes for the two indexes related to two randomized maternal treatments [52]. The weighted city-block distance was the combination of absolute values of the individual effect sizes for each index. As previously described, the weighting scheme was based on the assumption that the real effect size on each principal component factor was reflected by the excess of its Eigen value above the Kaiser criterion of 1 used in the PCA (ibid).

### Optical Recording of Olfactory Mucosal Activity

 In accordance with established procedures (e.g., 54-56), using optical methods and a voltage-sensitive dye we recorded the spatial patterns of neural activity across the olfactory epithelium in response to different odorants. After behavioral testing, rats were anesthetized and the right nasal cavity dissected exposing the flat septal epithelial surface. The tissue was next immersed in the voltage-sensitive dye di-4-ANEPPS as per protocol and later rinsed. In order to record from the tissue, the septal half of the nasal cavity was mounted in a Delrin chamber that contained a clear plastic window (e.g., 28). The septal mucosa was imaged onto a 640 x 480 pixel array of a Sony 14-bit CCD camera (Edmund Optics, Barrington, NJ). Uniformity in the placement of the septal images on the camera array was achieved by bringing each tissue into alignment with a “typical” septal outline.

 Airflow through the chamber was achieved through input and output ports that were in anatomical register with the tip of the nose and nasopharynx, respectively. For stimulation, a computer controlled negative pressure applied at the output port drew air or odorant across the septum (ibid). 

 Besides nicotine, we assessed the odorants carvone, heptanal, propyl acetate and ethylacetoacetate. The concentrations of the odorants were 0.40, 0.30, 0.04, 0.008 and 0.33, respectively (odorant concentrations are noted as the portion of vapor saturation at 23°C). Previous work has shown that these four stimuli (non-fetal- exposure odorants): (1) produced a different epithelial activity pattern in the rat (e.g., 55), (2) their patterns can be changed by salient experience [57] and (3) their odorant-specific spatial patterns of neural activity predict perception [54]. Amyl acetate (0.008) was used as an odorant standard.

 For each septum, recordings were done following the randomized presentation of the five odorants. Amyl acetate was presented at the beginning and end of the randomized odorant sequence.

 For every stimulus and every pixel of the camera array we recorded the neurophysiologic response. These response data were used to establish the differential spatial patterns of neural activity for the different stimuli and the peak magnitude of the overall neural response of the epithelium (e.g., 28,54)

 With respect to the first determination, several studies have shown that odorants create distinct spatial activity patterns across the olfactory epithelium (e.g., 57-59) and these patterns underlie the initial step in odorant quality coding [54,56]. To examine the effect of fetal nicotine exposure on these spatial activity patterns, we highlighted the regions of differential activity of each odorant by comparing it to the stimulus standard. That is, we subtracted, on a pixel-by-pixel basis, the equilibrated peak response of the individual odorants from the equilibrated response to the standard (e.g., 54,55,57) (N.B.: the process of equilibrating the array responses removes response magnitude as a confound in comparing patterns). For each odorant response recorded we then calculated the sum of the differences across the array of pixels, divided by the number of pixels and multiplied by 100. This value represented the average percent difference (i.e., APD) between each odorant and the amyl acetate standard for an individual animal.

 The peak magnitude of the epithelial response was determined by spatially averaging the response across the olfactory epithelium. To do this we: (1) summed the data recorded for each pixel of the array; (2) divided the summated data by the number of pixels; and (3) determined the peak height of the spatially averaged response. 

 All neurophysiologic data were analyzed with analysis of variance (ANOVA).

## Results

### Pilot Studies

#### Dose-Response

**Figure 1:**
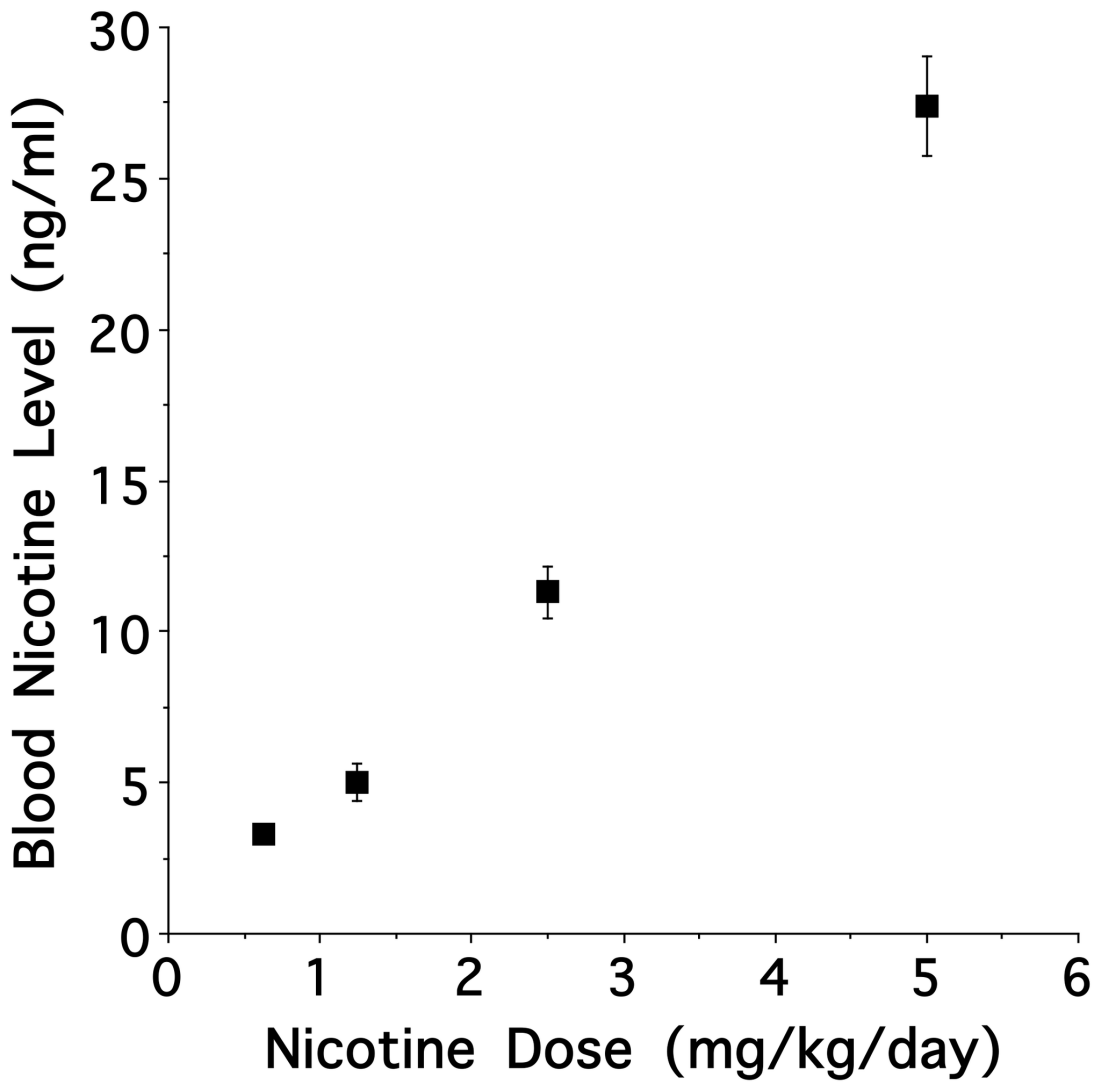
Blood nicotine levels as a function of mini-osmotic pump dose. The values illustrate the mean ± s.e.m.

As illustrated in Figure 1, trunk blood sampled from pregnant rat dams on G19 (two days before parturition) showed a significant linear relationship between nicotine dose delivered via mini-osmotic pump and blood nicotine levels (F [1, 20] = 326.58; *P* < 0.00001; R^2^ = 0.942). Importantly, on average, a dose of 5mg/kg/day yielded a blood nicotine level of 27±1.63ng/ml (mean±se).

#### Blood Nicotine Levels during Gestation

We next explored the stability of the 5mg/kg/day dose over time. Although dams gain a modicum of weight during the course of gestation there was relative stability of the blood nicotine levels. Linear regression analysis of blood nicotine data derived from dams sacrificed at G10, G13, G16 or G19 provided no evidence for a significant change in blood concentration (F [1, 20] = 0.16; *P* > 0.7; R^2^ = 0.007).

### Equivalence of Controls

 As noted in the *Methods* section, we included a non-surgical control (NP) along with a traditional vehicle loaded pump control (VC) in order to test directly whether the procedure of pump implantation and/or its physical presence during gestation had an untoward effect on our behavioral and/or neurophysiologic measures.

#### Behavioral Analysis

We tested the *a priori* hypothesis there would be no observable difference between VC vs. NP animals. In other words, that the weighted city-block distance of sized differences between the VC vs. NP control animals was zero in each of the three age groups. In this respect, we found no indication for a differential behavioral response between the two controls for either nicotine (t [27] = 1.02; *P* > 0.3) or EA odor (t [27] = 1.59; *P* > 0.1).

#### Neurophysiologic Analysis

Not surprisingly, in keeping with prior studies (e.g., 28,55,57), on average, there was a highly significant effect of odorant on both the spatial patterns of different odorants (F [4, 430] = 128.28; *P* < 0.00001) and the response magnitude (F [4, 437] = 117.26; *P* < 0.00001). Nonetheless, we found no observable difference between the VC vs. NP animals across the three ages in either neurophysiologic measure (F [1, 430] = 0.46; *P* > 0.50 and F [1, 437] = 0.07; *P* > 0.7, respectively).

 Given the foregoing findings of equivalency between the VC and NP animals, subsequent analyses with regard to specific effects of fetal nicotine experience versus control exposure were based on the use of combined controls (CT). In this respect, a single combined control group permitted a more tightly focused test of our *a priori* hypotheses while preserving any nominal average effect, if any, of both VC and NP exposures.

### Chemosensory Consequence of Fetal Nicotine Exposure

#### Behavioral Analysis

Our central focus was to determine whether fetal nicotine experience changes the innate behavioral responses of P17, P35 and P90 rats to nicotine odor across the life span. The primary test of an overall effect of maternal treatment (across all ages) involved the comparison of the weighted combination of effect sizes (in two dimensions) of prenatal nicotine vs. combined control exposures. The outcome of this principal analysis showed that, on average, there was a significant overall effect of prenatal nicotine exposure on the response to nicotine odor (t [27] = 2.56; *P* < 0.02). There was no evidence for an overall effect of sex (t [27] = 1.40; *P* > 0.17) but, nonetheless, we did find evidence for an overall sex by treatment interaction (t [27] = 2.17; *P* < 0.04).

**Figure 2:**
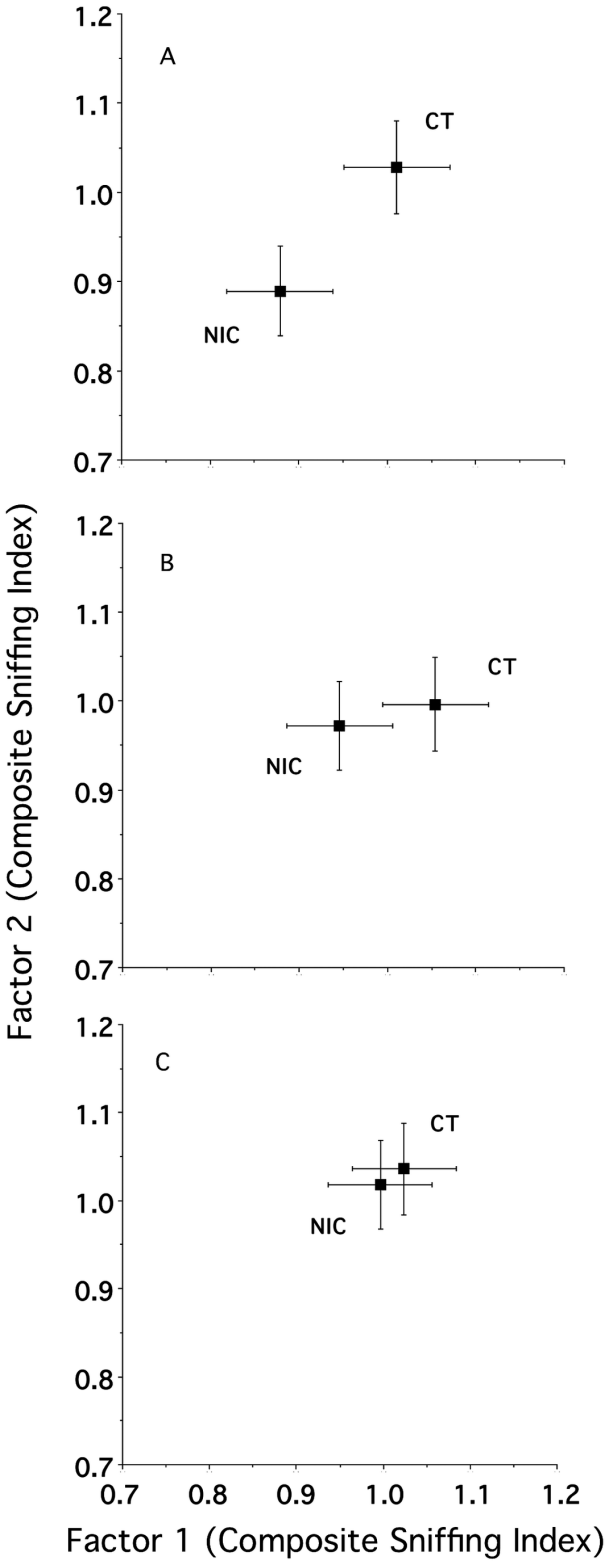
Composite sniffing indices for P17 (Panel A), P35 (Panel B) and P90 (Panel C) animals. The values illustrate the mean (± 2-dimensional s.e.m.) relative position of the nicotine versus combined controls in a stimulus-induced behavioral response space.

**Figure 3:**
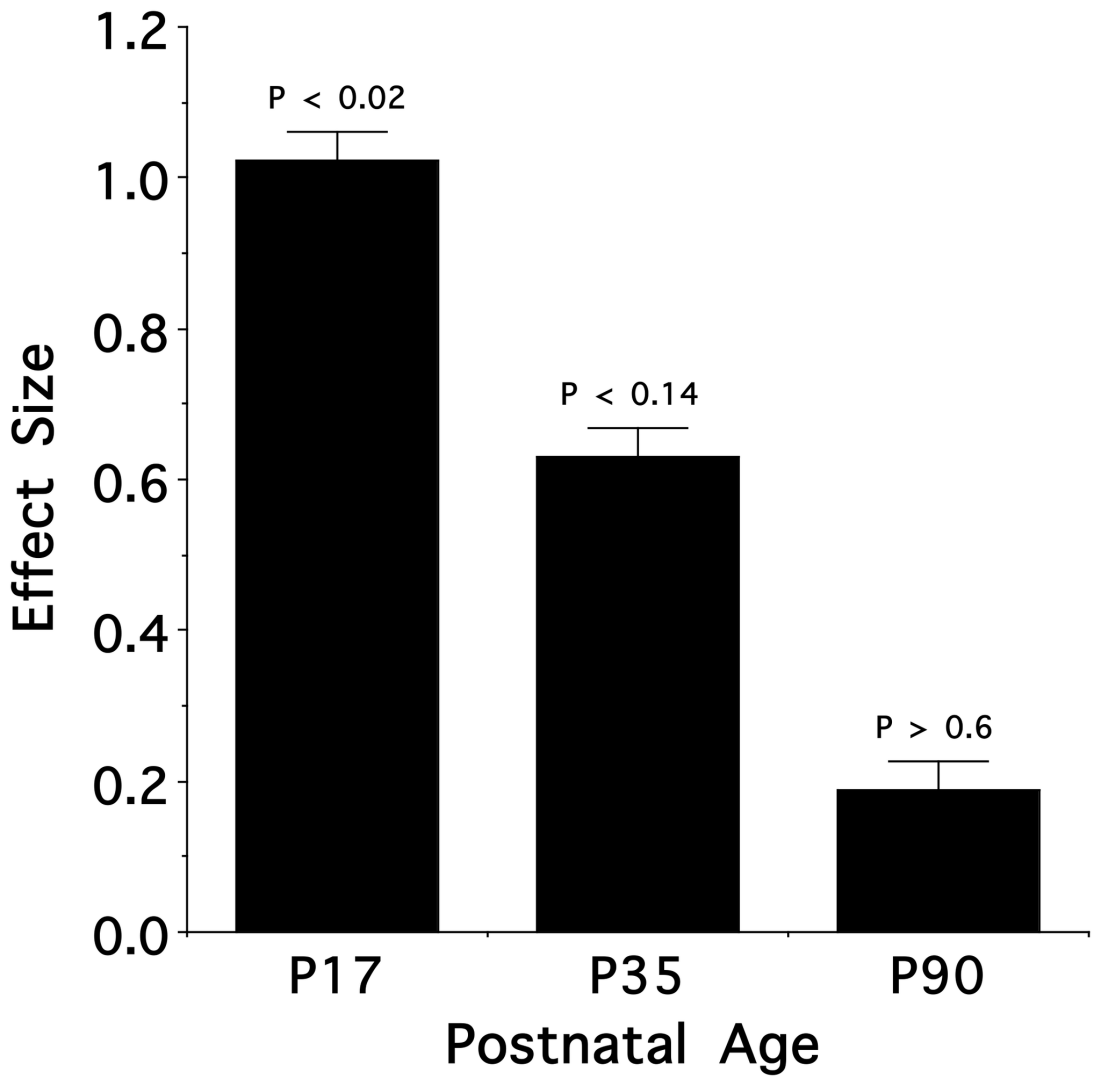
Nicotine weighted effect size (mean ± s.e.m) for P17, P35, and P90 ages. Relative to combined controls, the magnitude of the enhanced behavioral response of prenatal nicotine exposed animals to nicotine odor declined between P17 and P90.

 To give further interpretability to the above overall result we performed subsidiary assessments to explore the consequence of fetal experience at each age. Figure 2 (Panels A-C) illustrates the relative position (in two dimensions) of the prenatal nicotine versus combined control animals in a nicotine odor-mediated response space for each age. For each panel, the extent to which the animals’ responses to nicotine odor in each group were comparable maps as a level of proximity in the space (e.g., 27,29,30). As seen qualitatively in the three panels, the innate response to nicotine odor as a function of maternal treatment became more similar with age. Therefore, we next determined and tested the extent of the effect of prenatal nicotine versus combined control exposure at each individual age. As shown in Figure 3, the weighted city block distance between treatments means (NIC vs. CT), in two dimensions, (i.e., effect sizes) decreased with increasing age such that there was a significant effect at P17 (t [54] = 2.47; *P* < 0.02), an intermediate but non-significant effect at P35 (t [54] = 1.52; *P* < 0.14) and an amelioration of the effect at P90 (t [54] = 0.45; *P* > 0.6). Interestingly, for the P17 animals, relative to control exposure, the effect of fetal exposure was 4.7 times greater in the nicotine-exposed males as compared to the nicotine-exposed females (data not shown).

**Figure 4:**
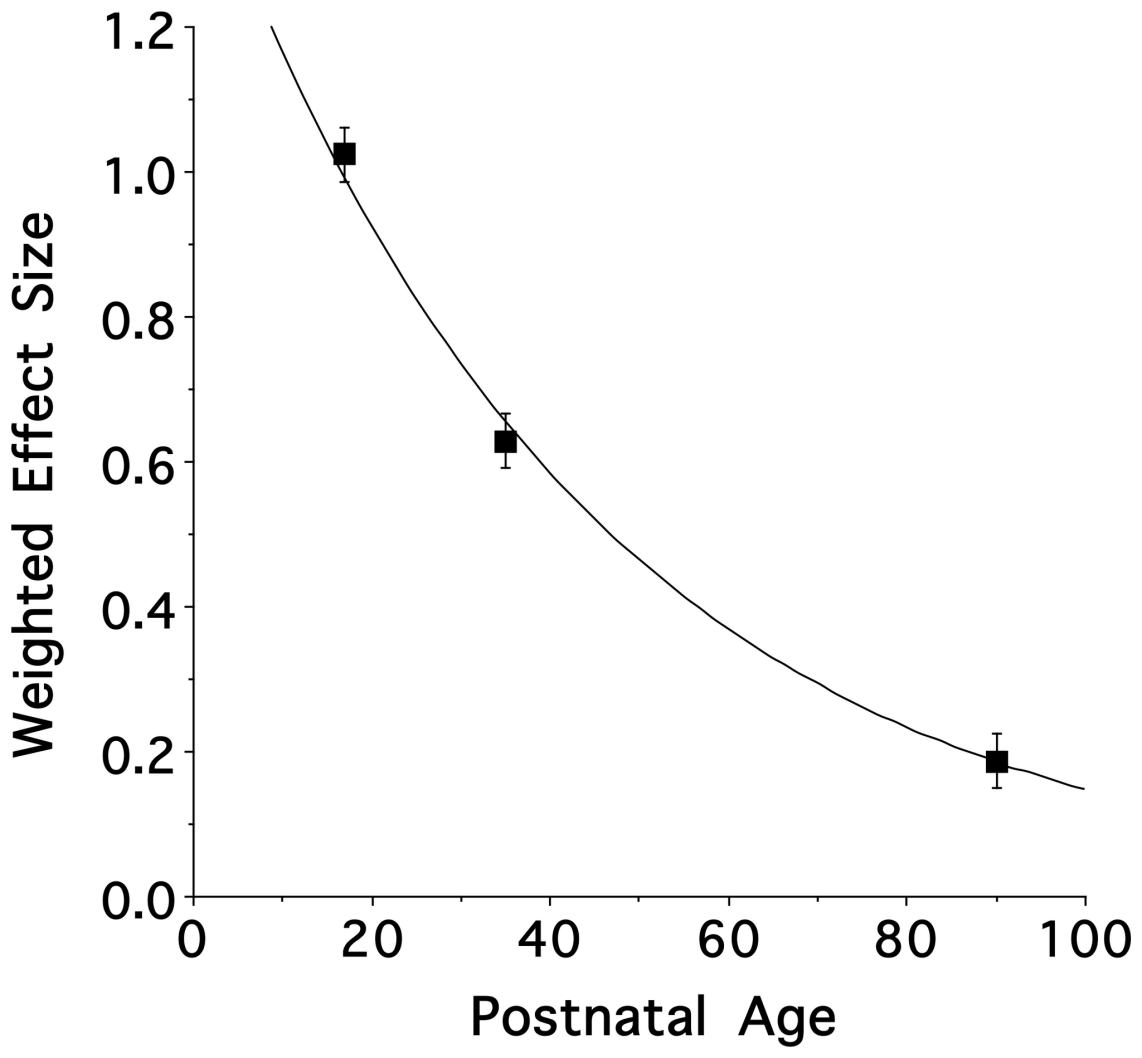
Ontologic nicotine odor response model of fetal exposure. The values illustrate the mean ± s.e.m. An exponential decay model accurately described the ontology of the postnatal behavioral response to nicotine odor.

 Examining the behavioral responses of early postnatal, adolescent and adult animals (Figure 3) allowed us to examine the ontogeny of the fetal exposure effect. Figure 4 illustrates that an exponential decay model accurately described the ontology of the postnatal behavioral response to nicotine odor. Importantly, the curve estimates that the window of increased responsiveness to nicotine odor encompasses a portion of the adolescent age range [37]. 

 To explore the specificity of the above a different group of P17, P35 and P90 animals were tested for their innate response to the non-fetal-exposure odorant EA. We found no indication for a general effect of maternal treatment (NIC vs. CT) on the animals’ responses to EA (t [27] = 1.22; *P* > 0.2). Moreover, we found no age-specific differences (all *P*’s > 0.08).

#### Neurophysiologic Analysis

Our assessment was directed toward testing, for each age, whether *in utero* nicotine exposure altered: (a) the spatially distinct differential activity patterns of the test odorants (note that, these patterns are the first stage in quality coding [54,56]); and/or (b) the overall responsiveness of the olfactory epithelium to nicotine odor as well as the four non-fetal exposure test odorants.

#### P17 rats.

**Figure 5:**
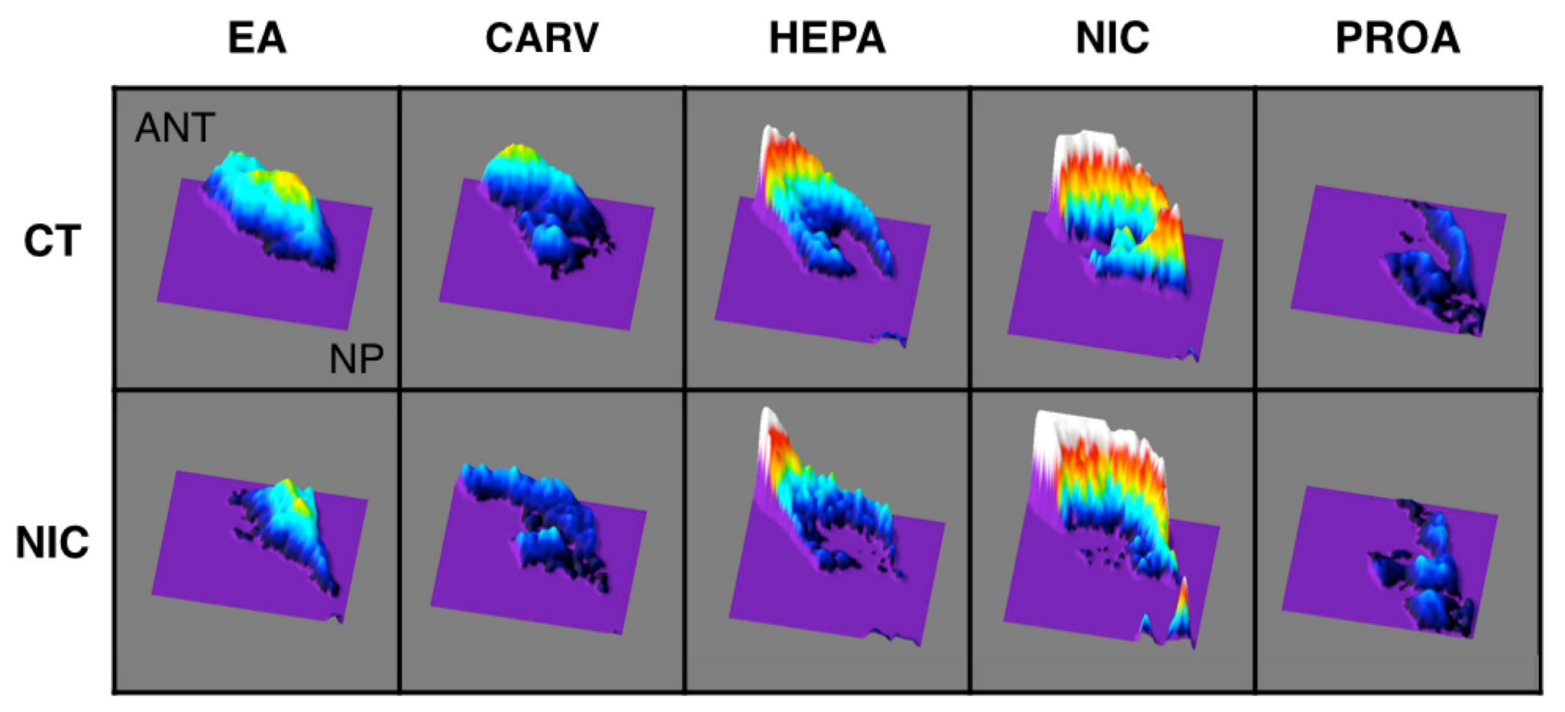
Average surface plots of the septal olfactory epithelium for P17 nicotine and combined controls maternal treatment group in response to ethylacetoacetate (EA), carvone (CA), heptanal (HEP), nicotine (NIC), and propyl acetate (PROA). The magnitude of the z-axis represents a shift in response for a specific stimulus at a specific pixel of the camera array compared with the standard for that same pixel. (N.B.: the entire array of responses has been equilibrated to a value of 100%). The point of reference of the response panels is: ANT = anterior and dorsal septum; NP = nasopharynx and ventral. NIC = fetal-nicotine-exposed rats; CT = combined VC and NP controls.

(a) *Odorant-Induced Activity Patterns*: With respect to the first question, as qualitatively highlighted in Figure 5, and in keeping with prior studies (e.g., 54-56,59), each odorant generated a unique spatial pattern of maximal activity (or “hot spot”). Importantly, despite the fact there were small differences across the individual animals in the shape of maximal differential odorant-induced activity the distinct location for each odorant was in a relatively comparable location of the olfactory epithelium for each rat (data not shown). In short, the location of unique spatial activity for each stimulus appeared comparable across all animals independent of maternal treatment. Nonetheless, the data in Figure 5 qualitatively suggest that the NIC vs. CT animals differed with regard to the extent of uniqueness in their differential response patterns. For example, relative to the odorant standard used in this study the region of comparative maximal activity for carvone appeared to be reduced in the fetal nicotine exposed animals whereas that to nicotine remained unchanged.

 To formally assess these qualitative observations, the uniqueness of each odorant’s differential spatial pattern of peak responses for each rat was evaluated. As expected (ibid), ANOVA demonstrated a highly significant effect of odorant stimulus on the APD (F [4, 239] = 65.24; *P* < 0.00001). Moreover, we found evidence for an overall effect of maternal treatment (F [1, 239] = 8.78; *P* < 0.004], with no evidence for an average effect of sex (F [1, 239] = 0.34; *P* > 0.5]) or treatment by odorant interaction (F [4, 239] = 0.99; *P* > 0.4]. Despite the lack of an average sex effect there was evidence for a sex by treatment interaction (F [1, 239] = 4.47; *P* < 0.04)

 Although the above analysis did not find evidence for a maternal treatment by odorant interaction, Figure 5 is clearly suggestive of such an effect. To evaluate this further, exploratory analyses were done to evaluate separately for a differential effect of maternal treatment on the response to nicotine odor and these same effects on the response to the other odorant stimuli. The results of these two analyses were consistent with our subjective impression of a preservation of the spatial response pattern to nicotine odor relative to the maternal treatment effects on the non-exposure odorants. In other words, on average, ANOVA found no differential effect of treatment, sex or sex by treatment interaction (all *Ps* > 0.4) on the response to nicotine odor. By contrast, for the non-exposure odorants there was strong evidence for an effect of treatment (F [3, 189] = 12.58, *P* < 0.0005) with no evidence of an effect of sex or treatment by odor interaction (all *Ps* > 0.13). There was evidence for a sex by treatment interaction (F [1, 189] = 6.19, *P* < 0.014).

#### (b): Responsiveness of the Olfactory Epithelium

Recall that, in evaluating the differential spatial activity patterns we equilibrated the array responses so that response size differences would not be confounded with our analyses of spatial pattern differences. Nonetheless, the overall neural responsiveness of the olfactory epithelium (question 2, above) represents a fundamental underlying element of the response to different odorants. Thus, in light of the above, we examined whether there was any difference in the effects of maternal treatment on the peak magnitude of the response to nicotine odor versus these same effects on the other odorants. We found no evidence for an overall effect of treatment, sex, or sex by treatment interaction on the peak magnitude of the response to nicotine odor (all *Ps* > 0.3). In contrast, for the non-fetal exposure odorants there was evidence for an average effect of prenatal treatment (F [1, 192] = 3.69, *P* < 0.05), sex (F [1, 192] = 17.67, *P* < 0.0001) and sex by treatment interaction (F [1, 192] = 13.53, *P* < 0.0003), with no evidence for an odorant by treatment interaction (F [3, 192] = 0.93, *P* > 0.4).

 The direction of the change in responsiveness to the non-exposure odorants was important to the interpretation of the foregoing result. Interestingly, on average, the ratio for the peak response magnitude was a 1.39-, 1.20-, 1.27- and 0.98-fold overall increase in response in the fetal nicotine treated animals for the odorants ethylacetoacetate, carvone, heptanal and propyl acetate, respectively.

#### P35 and P90

The data collected from the P35 and P90 animals was evaluated as described for the P17 rats. Here, our primary interest was to evaluate whether the early postnatal effect of fetal nicotine exposure persisted with the age of the animal.

#### (a): Odorant-Induced Activity Patterns

Consistent with the outcomes for the P17 rats, we found no indication for a change in the odorant-induced activity patterns in either age group. That is, although odorant stimulus significantly affected the pattern of differential mucosal activity (F [4, 204] = 96.61; *P* < 0.000001 and F [4,179] = 78.01; *P* < 0.000001: for P35 and P90, respectively), the distinguishing location of activity for each odorant was similar across maternal treatments for both ages (surface plots not shown). Nonetheless, for the P35 rats, there was no strong indication for an overall maternal treatment effect (F [1, 204] = 3.17; *P* >0.07), a significant sex effect (F [1, 204] = 3.73; *P* < 0.05) and no evidence for any first order interactions (all *Ps* > 0.55). For the P90 animals there was no evidence for any overall effect of treatment (F [2,179] = 0.39; *P* > 0.53), sex (F [1, 179] = 0.04; *P* > 0.82 or first order interactions (both *Ps* > 0.28).

#### (b): Responsiveness of the Olfactory Epithelium

In keeping with an amelioration of the maternal treatment effect on the odorant-induced mucosal activity patterns by P35, we found similar results for the overall responsiveness of the olfactory epithelium to the different odorants. In other words, for both the P35 and P90 age groups we found no strong evidence for an effect of maternal treatment, sex or sex by treatment interaction on the differential responsiveness to different odorants (all *Ps* > 0.08).

 In summary of our key neurophysiologic findings, we demonstrated that: (1) independent of maternal treatment the unique location of differential spatial activity for each odorant stimulus was comparable across all animals; (2) fetal nicotine exposure resulted in a stabilized magnitude of responsiveness and pattern distinctiveness in response to nicotine odor; and (3) fetal nicotine exposure resulted in an increase in responsivity and differential pattern muting in response to the non-exposure odorants. The effects of fetal exposure were ameliorated by P35.

## Discussion

 The human epidemiology suggests a contributory association between gestational nicotine experience via maternal smoking and the chance for becoming a smoker (e.g., 13,14). Moreover, the age of initial experience and the odds of continued nicotine abuse appear to be related [12,14]. Given the extent to which maternal tobacco use occurs during pregnancy (~ 25% [6]), understanding why the progeny of these mothers first start using tobacco products is critical to both prevention and timely treatment. To this point, a fundamental question arises - how does fetal exposure influence smoking and tobacco product acceptability and preference behavior?

 There is unmistakable data that olfactory plasticity serves to accentuate an animal’s attentiveness to odor stimuli that have “presumed” biological importance (e.g., 60,61). Indeed, there is a rich literature demonstrating that throughout development (namely, from the fetal period through adulthood) olfactory experience can influence: (1) later olfactory sensory function in terms of modulating, for example, intake and odor preferences (e.g., 33,62-71) and (2) the capacity to perceive and discriminate odors (e.g., 72-74). Of particular consequence to the present experiment is the general observation that fetal experience with an odorant can both change the neural sensitivity of the olfactory epithelium to the exposure odorant and modify postnatal acceptance and preference patterns (e.g., 33,63).

 Drugs of abuse such as nicotine are, themselves, odorants. Nicotine has an odor that, while described as sweet, warm and spicy, is also irritating and aversive at low to moderate concentrations [75-77]. Importantly, nicotine is known to have a substantial impact on the smell and flavor of tobacco smoke [78] and these sensory properties, in turn, prominently impact smoker pleasure [79]. As such, it is interesting to consider that prenatal experience with nicotine can, perhaps, increase its later acceptance through chemosensory mediated mechanisms. In this regard, important lessons have been learned from fetal exposure studies of another drug of abuse with aversive odor properties, namely, ethanol. Put broadly, these experiments showed a fundamental connection between olfactory function and postnatal avidity for ethanol as an outcome of fetal drug experience. P15 rats that experienced ethanol during gestation displayed a “tuned” or “focused” neural and behavioral response to the odor of ethanol that was specific to the drug [28]. Fetal exposure also yielded increases in ethanol intake [26]. Although the consequences of fetal exposure were gone in adults [26,28], the increased odor-meditated behavioral effects persisted into adolescence declining through young adulthood (e.g., 29,30,38). Notably, fetal experience with ethanol altered the olfactory system such that the aversive odor properties of the drug became more acceptable so as to enhance intake [27].

 In light of the above, the present experiments showed that nicotine exposure throughout gestation also yielded a change in olfactory function that included both behavioral and peripheral neural alterations in P17 rats and that these outcomes were completely absent in the fully mature animal. More specifically, compared with controls, animals that experienced nicotine throughout fetal development: (a) showed a preserved peripheral neural response to nicotine odor, while also demonstrating a generalized increase in responsiveness and loss in odorant-induced activity pattern distinctiveness (or pattern muting) to other odorant stimuli; (b) the neural consequence was marked at P17, being absent in P35 and P90 animals; (c) the observed neurophysiologic consequence in the early postnatal animal was paralleled by an enhanced innate behavioral response that was specific to nicotine odor; (d) however, the enhanced behavioral response was intermediate in extent in adolescent animals ameliorating completely by adulthood. Taken together, the current results are important in two basic ways. First, the present data highlight the generality of the chemosensory plasticity effect to another drug of potential abuse by demonstrating an association between fetal nicotine exposure and subsequent odor-guided behavioral and neural responsiveness to the drug. Second, in so doing, the data extend upon previous ethanol work by providing further evidence for the theoretical proposition there are experience-based chemosensory mechanisms by which a mother’s drug use can be passed to their progeny (e.g., 27).

 Regarding the above findings, a number of topics are important to consider. Here, we have taken liberty in labeling the behavioral valence of the fetal nicotine effect as a positive one (namely, either an enhanced response, or alternatively, a decreased aversion, to nicotine odor). To be sure, the plethysmography method, by itself, does not attribute a valence to any observed effect size between two groups. Even so, various data give support to our current interpretation: (a) contamination of the fetal environment with an innocuous odorant has uniformly been shown to yield increased intake and preference for the exposure stimulus (e.g., 33,62-69) and (b) using an identical behavioral testing paradigm, young rats exposed to fetal ethanol show an altered response to its odor that is causally linked to elevated ethanol avidity [27].

 With respect to the peripheral neural response, a fundamental issue is how the observed neural effects could underlie enhanced odor-mediated behavior. This is especially so in light of the observation that the peripheral neural effects ameliorated by adolescence. In keeping with studies on fetal ethanol [28,30], as well as other forms of long-term odor experience that increase the predilection for the exposure stimulus (e.g., 80,81) our data demonstrate that gestational nicotine stabilized the responsiveness to the nicotine odor against a backdrop of negative neural effects. Recall that, for the early postnatal animal: the neural responsiveness and distinctiveness of the odorant-induced activity pattern to nicotine odor was preserved in fetal exposed animals, whereas in these same animals there was an increase in responsiveness underlying a loss of pattern distinctiveness (or pattern muting) for the non-exposure odorants. This constellation of findings suggests a “focusing” of the nicotine encoding process at the level of the peripheral olfactory system and by extension the olfactory bulb. Different odorants induce distinct large-scale epithelial spatial patterns of sensory neuron activity (e.g., 55,59). Importantly, these patterns in turn constitute the first step in the encoding process (e.g., 54,56,82). Considering the stereotyped axonal targeting that occurs from the olfactory epithelium onto the olfactory bulb (e.g., 83), muting the differential patterns of glomerular activation would be expected to reflect a muting of these same odorant-induced patterns at the level of the periphery. This later point is important, as we would anticipate a stabilization of the key nicotine-encoding glomeruli to be essential to any process by which nicotine odor would increase associative meaning in the olfactory bulb. Indeed, the olfactory bulb is a key odor processing structure and, more importantly, it can encode that an odorant stimulus has gained associative importance (e.g., 31,84-87). How might this occur? Centrifugal inputs onto the olfactory bulb can impact context dependent alterations in olfactory bulb activity [88,89]. Further, there are specific central descending inputs onto the olfactory bulb that can modify odor responsiveness based on experience. For example, descending activity via monoaminergic and noradrenergic circuits indicating reward are required to alter the olfactory bulb’s response to odors that have gained significance (e.g., 90-93). Further to this point, recent evidence demonstrates that the connection between fetal ethanol-induced odor-mediated effects and postnatal avidity [27], requires the associative pairing between the odor quality of ethanol and its reinforcing properties [52].

 The foregoing discussion also establishes a framework for the absence of a connection between the adolescent behavioral and neural findings. Our data suggest that the odor-mediated behavioral effect outlasts the primary sensory neurons that were exposed to nicotine during fetal development. These neurons have turned over because there is a regular ongoing process of olfactory neuron cell death and regeneration in the epithelium (e.g., 94). Therefore, consistent with the above, while the peripheral neural effects of fetal nicotine exposure would be important in establishing the initial behavioral response it is not required for its preservation. In this regard, there is evidence that gestational ethanol exposure, for example, not only enhances the adolescent ethanol odor-mediated response, but also changes olfactory bulb genes involved in synaptic transmission and plasticity in addition to neuronal development [38]. 

 We also modeled the ontogeny of the behavioral odor-mediated consequence of prenatal nicotine exposure. The resulting mathematical model suggested an exponential decay curve with an enhanced nicotine odor response that, while falling within the range of ages defined as adolescence (N.B.: a vulnerable window for influencing long-term patterns of abuse of drug abuse [see rev [37].), did not persist beyond this intermediate period of development and into young adulthood. Thus, unlike with fetal ethanol exposure [30], postnatal susceptibility to the additive effects of pre- and postnatal nicotine experience may not endure over a considerable length of the animal’s life. Nonetheless, given that mothers who smoked during pregnancy are likely to continue during the nursing period and beyond, the period of vulnerability for the additive effects of pre-and post-natal is considerable.

## Conclusions

*In utero* nicotine exposure yielded an altered behavioral responsiveness to the odor of nicotine that was prominent in the early postnatal animal and, while evident in adolescence, the effect waned during this developmental period being completely ameliorated by adulthood. The intermediate behavioral effect during adolescence occurred without an increased response of the olfactory epithelium. The latter observation suggested that by P35 the olfactory epithelium returned to baseline function and that fetal nicotine exposure induced changes in olfactory bulb circuitry are likely responsible for the enhanced behavioral response. Taken together, the data provides evidence for an association between gestational nicotine experience and the behavioral responsiveness to the drug later in life that is altered, at least in part, by olfactory function. Importantly, the data add to a growing literature demonstrating experience-based chemosensory mechanisms by which a mother’s drug use can be transferred to their progeny.
